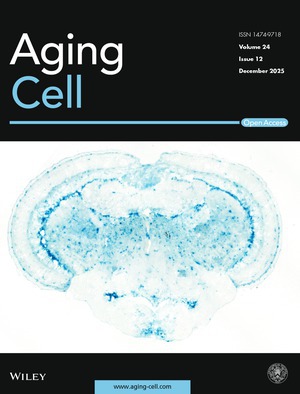# Additional Cover

**DOI:** 10.1111/acel.70323

**Published:** 2025-12-08

**Authors:** Raquel R. Martins, Savandara Besse, Pam S. Ellis, Rabia Sevil, Naomi Hartopp, Catherine Purse, Georgia Everett‐Brown, Owain Evans, Nadiyah Mughal, Mina H. F. Wahib, Zerkif Yazigan, Samir Morsli, Ada Jimenez‐Gonzalez, Andrew Grierson, Heather Mortiboys, Chrissy Hammond, Michael Rera, Catarina M. Henriques

## Abstract

Cover legend: The cover image is based on the article *Telomerase Depletion Accelerates Ageing of the Zebrafish Brain* by Raquel R. Martins et al., https://doi.org/10.1111/acel.70280.